# Immunohistochemical characterization of nodose cough receptor neurons projecting to the trachea of guinea pigs

**DOI:** 10.1186/1745-9974-4-9

**Published:** 2008-10-19

**Authors:** Stuart B Mazzone, Alice E McGovern

**Affiliations:** 1 School of Biomedical Sciences, The University of Queensland, St Lucia, 4072, Australia

## Abstract

**Background:**

Cough in guinea pigs is mediated in part by capsaicin-insensitive low threshold mechanoreceptors (cough receptors). Functional studies suggest that cough receptors represent a homogeneous population of nodose ganglia-derived sensory neurons. In the present study we set out to characterize the neurochemical profile of cough receptor neurons in the nodose ganglia.

**Methods:**

Nodose neurons projecting to the guinea pig trachea were retrogradely labeled with fluorogold and processed immunohistochemically for the expression of a variety of transporters (Na^+^/K^+^/2C1^- ^co-transporter (NKCC1), α1 and α3 Na^+^/K^+ ^ATPase, vesicular glutamate transporters (vGlut)1 and vGlut2), neurotransmitters (substance P, calcitonin gene-related peptide (CGRP), somatostatin, neuronal nitric oxide synthase (nNOS)) and cytosolic proteins (neurofilament, calretinin, calbindin, parvalbumin).

**Results:**

Fluorogold labeled ~3 per cent of neurons in the nodose ganglia with an average somal perimeter of 137 ± 6.2 μm (range 90–200 μm). All traced neurons (and seemingly all nodose neurons) were immunoreactive for NKCC1. Many (> 90 per cent) were also immunoreactive for vGlut2 and neurofilament and between 50 and 85 per cent expressed α1 ATPase, α3 ATPase or vGlut1. Cough receptor neurons that did not express the above markers could not be differentiated based on somal size, with the exception of neurofilament negative neurons which were significantly smaller (P < 0.05). Less than 10 per cent of fluorogold labeled neurons expressed substance P or CGRP (and these had somal perimeters less than 110 μm) and none expressed somatostatin, calretinin, calbindin or parvalbumin. Two distinct patterns of nNOS labeling was observed in the general population of nodose neurons: most neurons contained cytosolic clusters of moderately intense immunoreactivity whereas less than 10 per cent of neurons displayed uniform intensely fluorescent somal labeling. Less than 3 per cent of the retrogradely traced neurons were intensely fluorescent for nNOS (most showed clusters of nNOS immunoreactivity) and nNOS immunoreactivity was not expressed by cough receptor nerve terminals in the tracheal wall.

**Conclusion:**

These data provide further insights into the neurochemistry of nodose cough receptors and suggest that despite their high degree of functional homogeneity, nodose cough receptors subtypes may eventually be distinguished based on neurochemical profile.

## Background

Previous studies have identified a novel vagal sensory nerve subtype that innervates the large airways (larynx, trachea and main bronchi) of guinea pigs and is likely responsible for defensive cough in this species [1]. These sensory neurons (referred to as cough receptors) are derived from the nodose ganglia and are characterized by their insensitivity to capsaicin and their sensitivity to both rapid reductions in pH and punctuate (touch-like) mechanical stimulation [1-3]. However, unlike other classically defined low threshold mechanoreceptors which innervate the airways and lungs, cough receptors display a low sensitivity to mechanical stretch (including inflation/deflation and bronchospasm), conduct action potentials slower (~5 m/sec for cough receptors compared to > 15 m/sec for intrapulmonary stretch receptors) and are unresponsive to the purinergic agonist α,β-methylene ATP [1]. Based on these observations, cough receptors are believed to represent a distinct airway afferent nerve in this species (reviewed in [4]).

Functional and electrophysiological studies have provided key insights into the role of nodose cough receptors in the cough reflex. In anesthetized guinea pigs, punctuate mechanical stimulation or rapid acidification of the laryngeal or tracheal mucosa evokes coughing, a response that can be abolished by selectively disrupting the afferent pathways from the nodose ganglia [1,5-7]. Extensive electrophysiological analyses of the activation profiles of nodose neurons projecting to the guinea pig trachea and larynx suggests that the majority (perhaps greater than 95%) of these neurons form a seemingly homogeneous population of neurons that display the functional characteristics of cough receptors [1,2]. In the guinea pig trachea and larynx, there are very few nodose capsaicin-sensitive nociceptors (tracheal nociceptors are mostly derived from the jugular vagal ganglia) and no classically defined rapidly adapting or slowly adapting stretch receptors [1,2].

**Figure 1:**
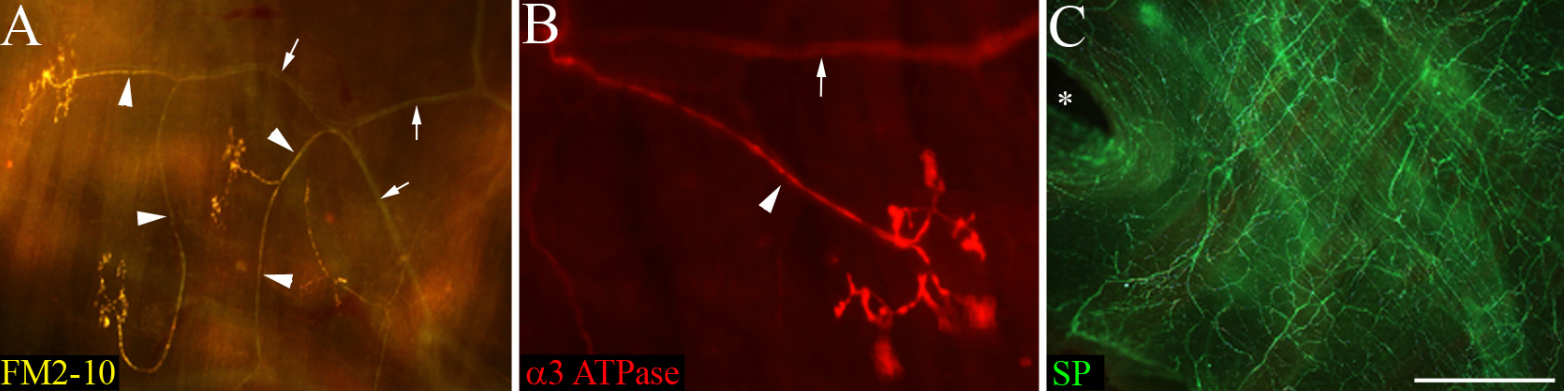
**Morphology of cough receptor nerve terminals in the guinea pig trachea.** Presumed cough receptor nerve terminals labeled (A) with the vital styryl dye FM2-10 and (B) immunohistochemically for α3 Na+/K+ ATPase. Note the terminal structures are arranged parallel to the tracheal muscle fibers (running from top to bottom of the panels). The cough receptor terminals (A, B) are clearly differentiated from substance P-containing (SP) tracheal nociceptors (C). The arrow heads and small arrows in panels (A) and (B) illustrate individual cough receptor axons and the nerve bundles from which the axons arise, respectively. The asterisk in panel (C) shows the origin of a primary bronchus at the caudal end of the trachea. The scale bar represents 200 μm in panel (A) and 50 μm in panels (B) and (C). These images were generated, but not used for publication, during previous studies (FM2-10 staining from reference [9] and α3 Na+/K+ ATPase/SP immunohistochemistry from reference [6]). Refer to [6,9] for detailed methods.

Anatomical and immunohistochemical studies have also provided some information about the nodose cough receptor. In the tracheal wall, the peripheral terminals of mechanoreceptors (presumably cough receptors) have been differentiated from substance P expressing nociceptors using osmium staining techniques [8], the intravital styryl dye FM2-10 [7,9], as well as with immunostaining for the alpha3-expressing isozymes of Na+/K+ ATPase and the furosemide sensitive Na+/K+/2Cl- co-transporter NKCC1 [6] (see also Fig 1). Retrograde labeling of afferents innervating the guinea pig trachea have shown that the majority of tracheal nodose neurons express neurofilament proteins (associated with myelinated neurons) but are devoid of the neuropeptides substance P, CGRP and the capsaicin receptor TRPV1 (all associated with capsaicin-sensitive sensory nerves) [2,10,11]. These observations would also support the suggestion that most nodose neurons innervating the guinea pig trachea and larynx are cough receptors and that these cough receptor neurons may be a homogeneous population in the nodose ganglia. However, a detailed neurochemical profile of these neurons has not been performed and as such, the possibility of cough receptor heterogeneity cannot be excluded.

Immunohistochemical studies of other sensory nerve populations have successfully used the expression of proton pump isozymes, vesicular glutamate transporters (vGluts; a marker for glutamatergic neurons), neuropeptides and calcium binding proteins (such as calretinin, calbindin and parvalbumin) as useful markers for characterizing sensory nerve subtypes. Therefore, in the present study we used well characterized antisera raised against these transporters, neurotransmitters and cytosolic proteins to further characterize the guinea pig cough receptor neurons in the nodose ganglia.

## Methods

Experiments were approved by the Howard Florey Institute Animal Ethics Committee and conducted on male albino Hartley guinea pigs (200–350 g, n = 36, IVMS, South Australia) at the Howard Florey Institute (The University of Melbourne, Australia).

### Retrograde tracing

Guinea pigs (n = 32) were anesthetized with 1.8–2.2 per cent isoflurane in oxygen. The extrathoracic trachea was exposed via a ventral incision in the animal's neck. Using a 10 μl Hamilton glass microsyringe fitted with a 32 gauge needle, 10 μl of 4 per cent fluorogold (Fluorochrome LLC, Colorado, USA) was injected into the rostral extrathoracic tracheal lumen (on to the mucosal surface). Following injection, the wound was sutured and the animals were allowed to recover for 7 days at which time they were anesthetized with sodium pentobarbital (100 mg/kg i.p.) and transcardially perfused with 10 mM phosphate buffered saline (PBS) followed by 4% paraformaldehyde in PBS. The nodose ganglia was removed and placed in 4% paraformaldehyde at 4°C for 2 hours, then cyroprotected in 20% sucrose solution at 4°C overnight prior to immunohistochemical processing (see below).

### Preparation of tracheal wholemounts

Wholemount preparations of guinea pig (n = 4) tracheal segments were prepared using a modification of previously described methods [6,8]. Briefly, animals were deeply anesthetized with sodium pentobarbital (80 mg/kg i.p) and transcardially perfused with 500 mL of 10 mM phosphate buffered saline (PBS). The entire trachea was removed, cleaned of excess connective tissue, and opened longitudinally via a midline incision along the ventral surface. The epithelium was gently rubbed off the trachea with a cotton swab and tracheal segments (8–10 rings in length) were pinned flat onto a piece of cork board and placed in fixative (4% paraformaldehyde) for 2–3 hours at 4°C, and then transferred to blocking solution (10 mM PBS and 10% horse serum) for one hour prior to immunohistochemical staining (see below). Epithelial removal is necessary to visualize cough receptor nerve terminals in the guinea pig trachea which are confined to the extracellular matrix below the epithelium. This procedure would be expected to remove some tracheal nociceptors [8] but does not disrupt cough receptors [7,9].

### Immunohistochemistry and microscopy

**Figure 2:**
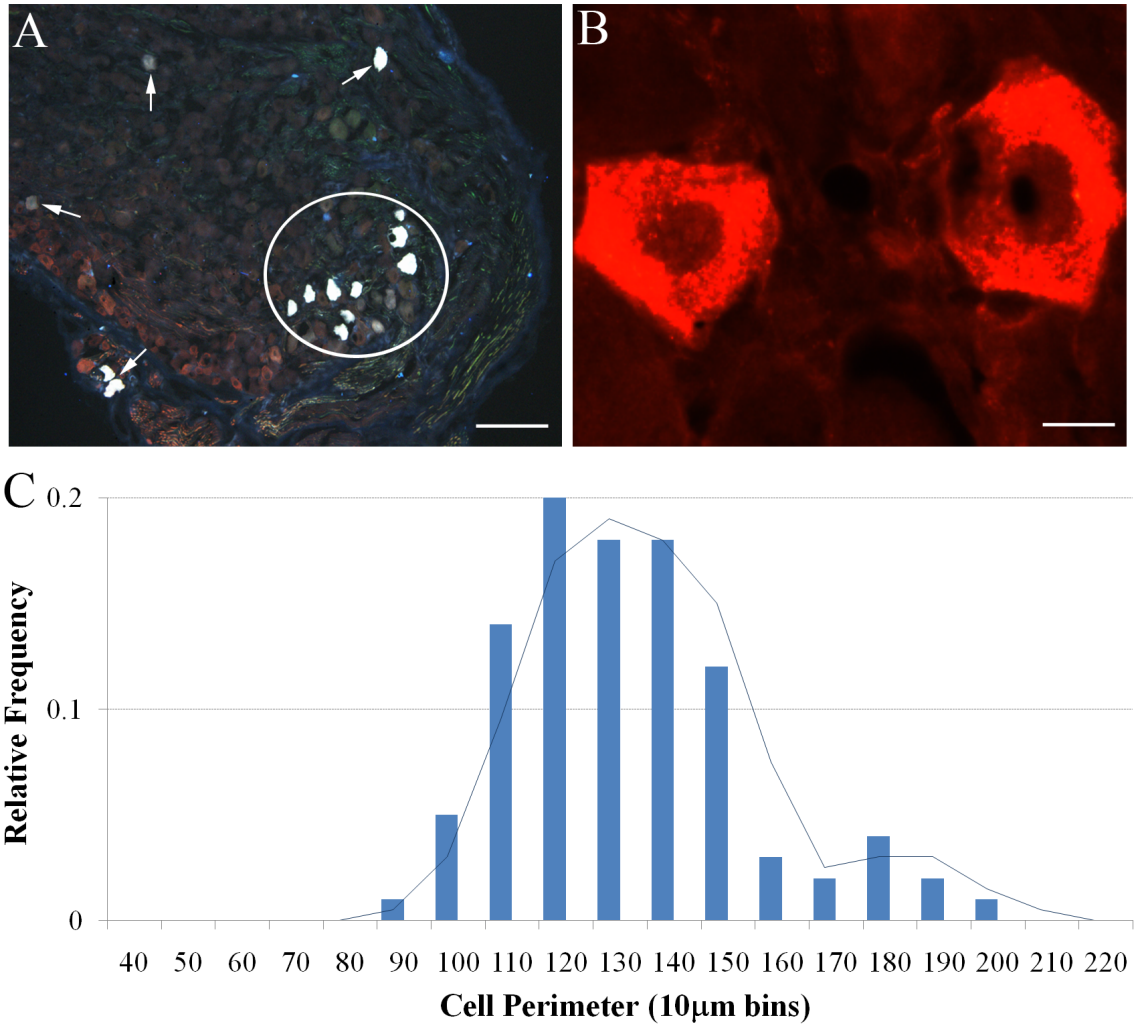
**Retrograde labeling of nodose neurons innervating the guinea pig trachea.** (A) Low magnification of nodose ganglia showing individual (arrows) and clusters (circle) of fluorogold labeled nodose neurons that have not undergone subsequent secondary immunoprocessing (hence the neurons appear blue). (B) Higher magnification of two fluorogold-labeled nodose neurons that have undergone secondary immunoprocessing and relabeled with a rhodamine fluorophore (hence the neurons appear red). See methods for details. Scale bars represent 150 μm in A and 20 μm in B. (C) Histogram showing the distribution of retrogradely labeled nodose neurons based on somal perimeter. The superimposed line graph shows the moving average calculated from the histogram. See text for details.

Immunohistochemical staining was performed as previously described [6]. Briefly, nodose ganglia were rapidly frozen in OCT embedding media, and 16 μm cryostat-cut sections were mounted directly onto subbed glass slides. Slides were incubated for 1 hour in blocking solution (10% horse serum), and then overnight (at room temperature) in PBS/0.3% Triton X-100/2% horse serum along with the primary antisera of interest (Table 1). Sections were washed several times with PBS, and then incubated with the appropriate AlexaFluor-conjugated secondary antisera (Table 1). All sections were cover-slipped with buffer glycerol immediately prior to microscopy. In some instances, fluorogold was found to be rapidly quenched during microscopy making accurate cell counting and photography difficult. On these occasions, coverslips were removed and the sections were incubated with rabbit anti-fluorogold (1:10,000; Fluorochrome LLC, Colorado, USA), followed by AlexaFluor 594-congugated donkey anti-rabbit antibodies (Table 1). Accordingly, some fluorogold cells shown in the representative photomicrographs appear blue (when quenching was not a problem) and others appear red (when stabilized with secondary immunoprocessing processing) (see Fig 2 for example).

**Table 1:** Details of the primary and secondary antibodies used for immunohistochemical staining.

	Host	Dilution	Source
*Primary Antibodies – Transporters*			
α1 Na+/K+ ATPase (clone 05–369)	Mouse	1:100	Millipore, Australia.
α3 Na+/K+ ATPase (clone XVIF9-G10)	Mouse	1:400	Biomol, PA, USA.
NKCC1	Rabbit	1:1000	Gift Dr RJ Turner, National Institute of Dental and Craniofacial Research, USA.
vGLUT1 (catalogue# 135 302)	Rabbit	1:2000	Synaptic Systems Goettingen, Germany.
vGLUT2 (catalogue# 135 402)	Rabbit	1:2000	Synaptic Systems Goettingen, Germany.
*Primary Antibodies – Neurotransmitters*			
CGRP (catalogue# RPN 1842)	Rabbit	1:4000	Amersham, UK.
Neuronal nitric oxide synthase (nNOS)	Sheep	1:4000	Gift Dr Colin Anderson, University of Melbourne, Australia.
Somatostatin (catalogue# AB5494)	Rabbit	1:100	Millipore, Australia.
Substance P (clone NC1)	Rat	1:200	Millipore, Australia.
*Primary Antibodies – Cytosolic Proteins*			
Calbindin D28k (number CB-38A)	Rabbit	1:1000	Swant Bellinzona, Switzerland.
Calretinin (number 7699/4)	Rabbit	1:1000	Swant Bellinzona, Switzerland.
Neurofilament 160KD (clone NN18)	Mouse	1:400	Millipore, Australia.
Parvalbumin (number 235)	Mouse	1:400	Swant Bellinzona, Switzerland.
*Secondary Antibodies (IgG, H+L, 2 mg/ml)*			
AlexaFluor 488 or 594 anti-goat	Donkey	1:200	Molecular Probes Eugene, OR, USA
AlexaFluor 488 or 594 anti-mouse	Donkey	1:200	Molecular Probes Eugene, OR, US
AlexaFluor 488 or 594 anti-rabbit	Donkey	1:200	Molecular Probes Eugene, OR, USA
AlexaFluor 488 anti-rat	Goat	1:200	Molecular Probes Eugene, OR, USA

*Note*, AlexaFluor 488 and 594 are green and red fluorphores, respectively.

Immunohistochemical processing of tracheal wholemounts was performed using a modification of the methods described above for nodose sections. Tissues were first pinned flat to a sylgard-filled tissue culture dish and incubated for 1 hour in blocking solution (10% normal horse serum in 10 mM PBS) and then overnight (at 37°C) in 10 mM PBS/0.3% Triton X-100/2% horse serum containing the primary antisera of interest (refer Table 1). After washing thoroughly with 10 mM PBS (for at least 3 hours), wholemounts were then incubated for 1 hour at room temperature in the appropriate AlexaFluor-conjugated secondary antibody (refer Table 1).

Labeling of wholemounts and slide mounted sections was visualized using an Olympus BX51 fluorescent microscope equipped with appropriate filters and an Optronics digital camera. Low and high magnification images were captured and stored digitally for subsequent off-line analysis of somal size (see below) and preparation of representative photomicrographs. Negative control experiments, in which the primary antisera were excluded, were carried out where necessary.

### Data analysis

Cell counts in a given field of view were performed either online (during microscopy) or offline (using high resolution digital images) at 100–200× magnification. 3–10 representative replicate sections were assessed per animal and a minimum of 4 animals were analyzed per group. For somal size analysis, stored images were imported into ImageJ software (NIH, USA ) and cell edges were traced on screen using a calibrated scale tool. Only cells with a distinct nuclear region were measured in order to increase the likelihood that perimeters were measured close to the middle of the neuron and therefore accurately reflected the true somal size. A minimum of 100 labeled cells, taken from at least 3 different animals, were used to estimate somal sizes for each marker. Data are expressed as a mean ± SEM. Differences between group data are compared using a Student's t-test and significance was set at P < 0.05.

## Results

### Fluorogold retrograde labeling

**Figure 3:**
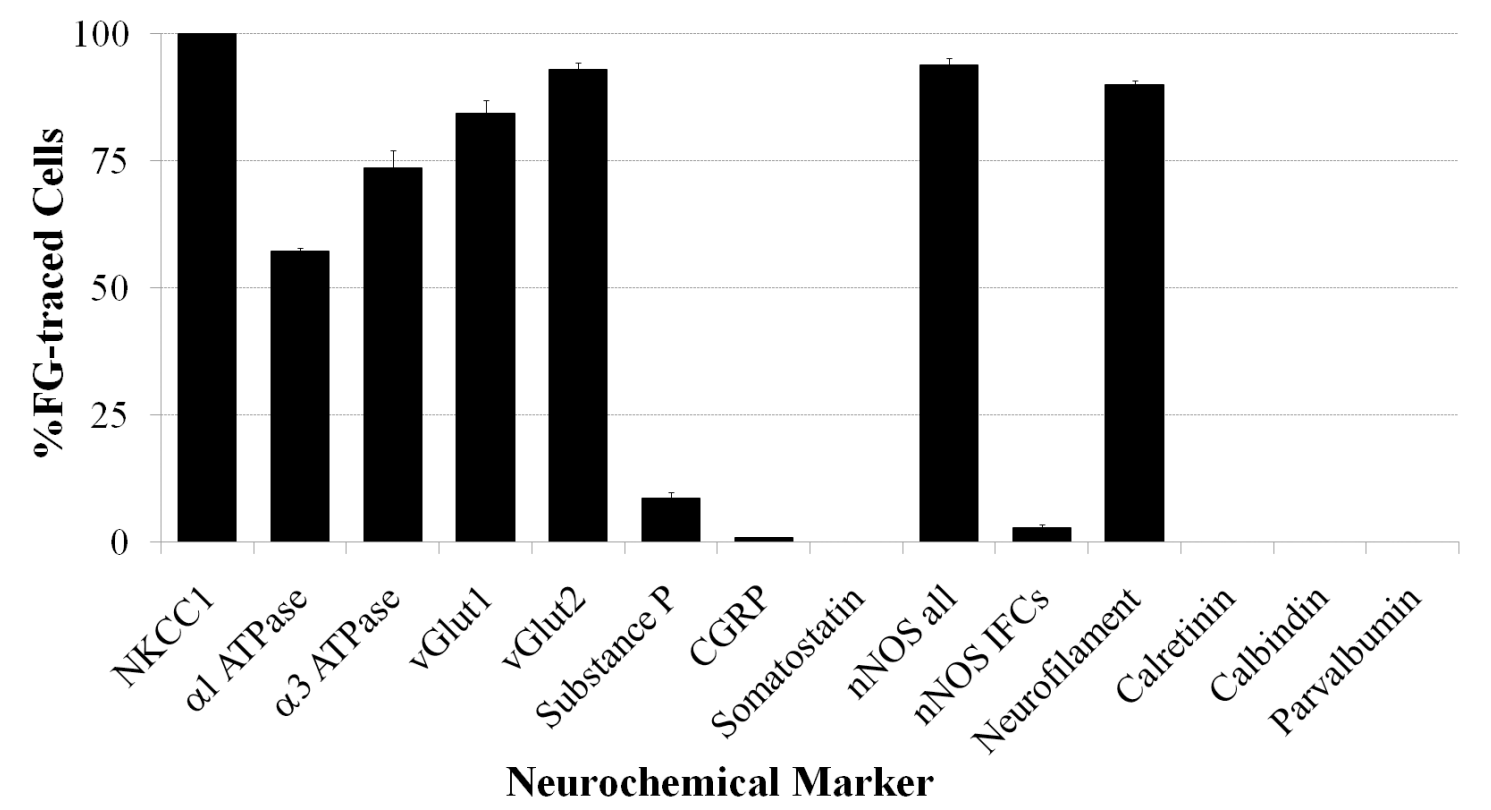
**Summary of the neurochemical profile of retrogradely labeled nodose neurons.** The data represent the mean ± SEM (minimum 3 nodose sections from n = 4–5 animals) per cent of fluorogold (FG) traced neurons that stained positive for the neurochemical markers. Explanation of neurochemical marker labels: *NKCC1*, Na+/K+/2Cl- co-transporter 1; *vGlut*, vesicular glutamate transporter; *CGRP*, calcitonin gene-related peptide; *nNOS all*, all cells expressing detectable neuronal nitric oxide synthase; *nNOS IFCs*, nNOS Intensely fluorescent cells.

Injection of fluorogold into the rostral trachea labeled neurons bilaterally in the nodose ganglia (Fig 2A, B). In 4 experiments, fluorogold labeled neurons represented 2.8 ± 0.4 per cent of the total cell population (assessed using NKCC1 immunoreactivity as a pan-neuronal marker, see below). As previously reported, retrogradely labeled soma appeared randomly distributed throughout the ganglia with no obvious topographical organization [2,11]. Most (> 80 percent) traced neurons had somal perimeters ranging between 100–150 μm (average 137.3 ± 6.2 μm), although neurons as small as 90 μm and up to 200 μm in size were less frequently noted (Fig 2C). The percentage of fluorogold traced neurons expressing each of the immunohistochemical markers tested is summarized in Fig 3 and discussed in more detail below.

### Immunohistochemical expression of transporter proteins in nodose ganglia

**Figure 4:**
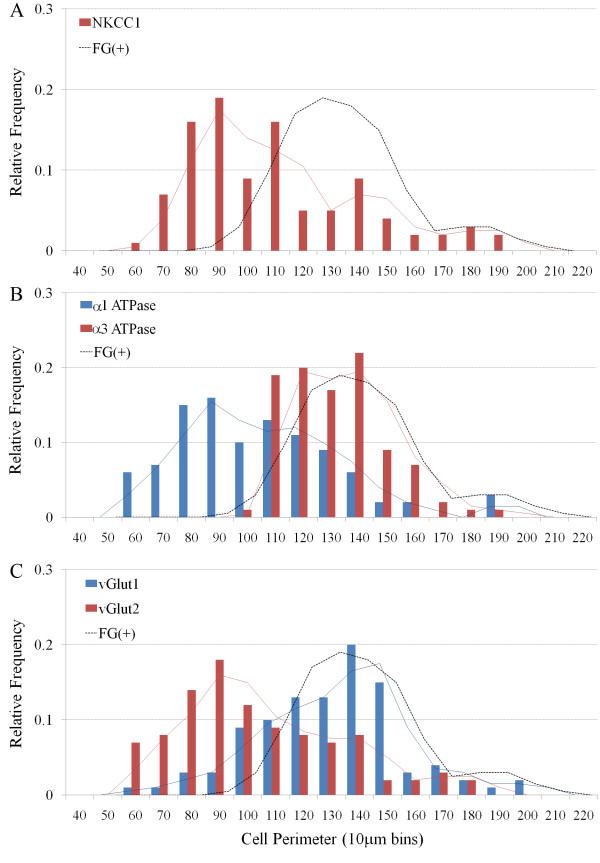
**Histograms showing the size distribution of all nodose neurons (irrespective of fluorogold tracing) that express (A) NKCC1, (B) α1 Na+/K+ ATPase or α3 Na+/K+ ATPase, and (C) vGlut1 or vGlut2.** The superimposed solid lines show the moving averages associated with each histogram and the dashed line references the size distribution of fluorogold (FG) traced neurons shown in figure 2.

**Figure 5:**
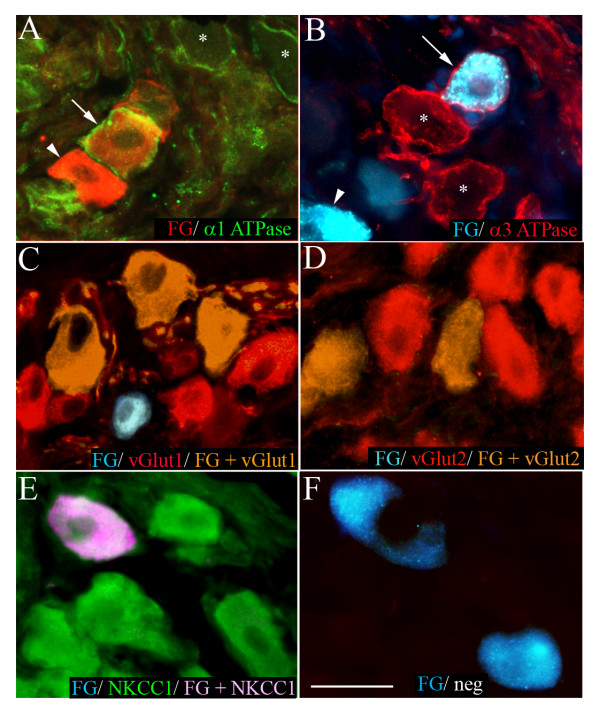
**Representative photomicrographs showing nodose neurons retrogradely labeled from the trachea with fluorogold (FG) overlaid with immunoreactivity for (A) α1 Na+/K+ ATPase, (B) α3 Na+/K+ ATPase, (C) vGlut1, (D) vGlut2, (E) NKCC1 or (F) negative control (neg). **In panels A and B, the arrows point to FG traced neurons that are immunoreactive for α1 or α3 Na+/K+ ATPase, the arrow heads show traced neurons that are not immunoreactive for α1 or α3 Na+/K+ ATPase and the asterisks show FG-negative neurons that are immunolabeled for α1 or α3 Na+/K+ ATPase. Traced neurons appear red in panel A as the tissue underwent secondary immunoprocessing for FG. Scale bar represents 40 μm.

**Figure 6:**
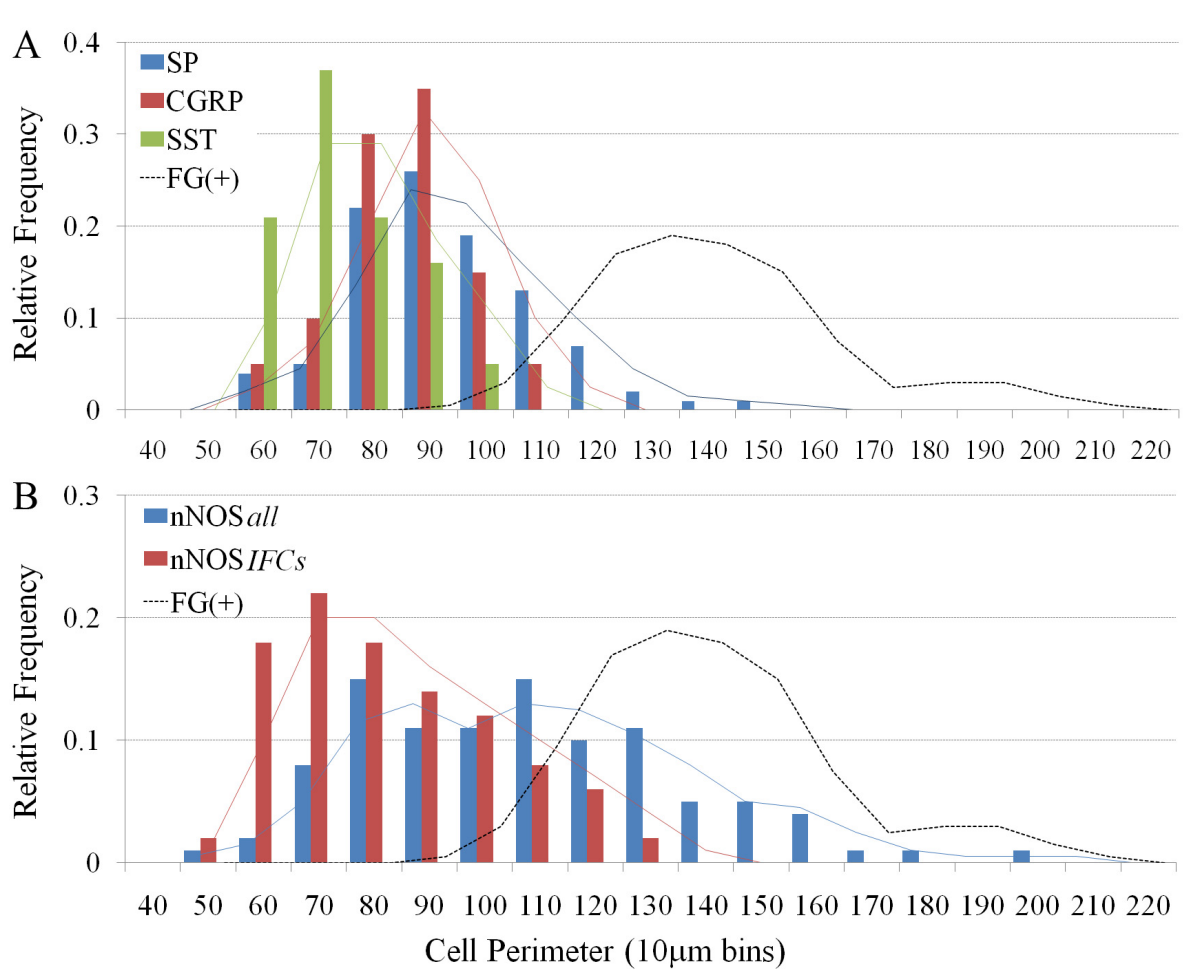
**Histograms showing the size distribution of all nodose neurons (irrespective of fluorogold tracing) that express (A) substance P (SP), calcitonin gene-related peptide (CGRP) or somatostatin (SST), and (B) neuronal nitric oxide synthase (nNOS).** In panel B nNOS *all *denotes all nNOS immunoreactive cells whereas nNOS *IFCs *denotes only nNOS intensely fluorescent cells. The superimposed solid lines show the moving averages associated with each histogram and the dashed line references the size distribution of fluorogold (FG) traced neurons shown in figure 2.

NKCC1 immunoreactivity was present in neurons from a wide range of somal sizes (ranging from 60–200 μm, average 113.9 ± 3.1 μm) (Fig 4A) and likely represents a pan-neuronal marker for vagal sensory neurons (Fig 3; [6]). By contrast, α1 and α3 Na+/K+ ATPase, vGlut1 and vGlut2 immunoreactivity was not universally expressed by all neurons in the nodose ganglia (data not directly shown but can inferred from Fig 3). Both α1 Na+/K+ ATPase and vGlut2 immunoreactivity was present in cells with a large range of somal sizes (60–190 μm, average 109.3 ± 2.8 μm and 109.6 ± 3.0 μm, respectively), whereas α3 Na+/K+ ATPase and vGlut1 immunoreactivity was primarily limited to medium and larger sized neurons (100–190 μm, average 139.1 ± 1.8 μm and 135.7 ± 2.7 μm, respectively) (Fig 4B, C). The pattern of labeling observed for the various transporter markers also varied. NKCC1, vGlut1 and vGlut2 immunoreactivity was found throughout the cytoplasm while α1 and α3 Na+/K+ ATPase immunoreactivity was principally confined to the cell membrane (Fig 5).

NKCC1 immunoreactivity was present in all retrogradely labeled neurons that were assessed for this marker (Fig 3 and Fig 5). The vast majority (84–93 percent) of traced neurons were also immunoreactive for vGlut1 or vGlut2 and many (57–73 per cent) expressed α1 or α3 Na+/K+ ATPase on their plasma membranes (Fig 3 and Fig 5). Those populations of neurons that were retrogradely labeled by fluorogold but did not show immunoreactivity for the relevant transporter markers did not significantly differ in size from the overall population of traced neurons (Table 2) and showed no other obvious morphological characteristics that would differentiate them from the population of traced cells that expressed the marker. Exclusion of the primary antisera prevented detectable immunoreactivity in all cases (for example, Fig 5F).

**Table 2:** Mean cell sizes of guinea pig nodose neurons.

**Markers ** **Commonly ** **Expressed**^1^	**Average Cell Perimeter (μm) **		**Markers ** **Uncommonly ** **Expressed**^2^	**Average Cell Perimeter (μm) **	
	***Marker (+)***	***Marker(-)/FG(+)***		***Marker (+)***	***Marker(+)/FG(+)***
NKCC1	113.9 ± 3.1*	None	Substance P	99.1 ± 3.7*	108.3 ± 5.9*
α1 Na^+^/K^+ ^ATPase	109.3 ± 4.8*	131.3 ± 8.4	CGRP	90.0 ± 2.6*	92.9^3 ^*
α3 Na^+^/K^+ ^ATPase	139.1 ± 1.8	128.5 ± 5.1	Somatostatin	80.4 ± 2.2*	None
vGlut1	135.7 ± 2.6	126.8 ± 4.7	nNOS *(IFCs)*	98.3 ± 2.6*	109.5 ± 8.9*
vGlut2	109.6 ± 3.0*	138.6 ± 1.8	Calretinin	177.9 ± 2.6*	None
Neurofilament	142.1 ± 5.7	105.3 ± 2.4*	Calbindin	173.3 ± 2.1*	None
nNOS *(all)*	112.6 ± 2.9*	126.8 ± 5.9	Parvalbumin	None	None

^1 ^Defined as a marker that is expressed in more than 50 per cent of the FG traced neurons.

^2 ^Defined as a marker that is expressed in less than 50 per cent of the FG traced neurons.

^3 ^Only one FG traced neuron expressed CGRP.

*P < 0.05, significantly different compared to the average size of FG traced neurons, Student's t-test (*Note*. The average somal perimeter of FG traced neurons = 137.3 ± 6.2).

Abbreviations: CGRP; Calcitonin Gene-Related Peptide; FG, Fluorogold; IFCs, Intensely Fluorescent Cells; nNOS, neuronal Nitric Oxide Synthase; vGlut, vesicular Glutamate transporter.

### Immunohistochemical expression of neurotransmitters in nodose ganglia

**Figure 7:**
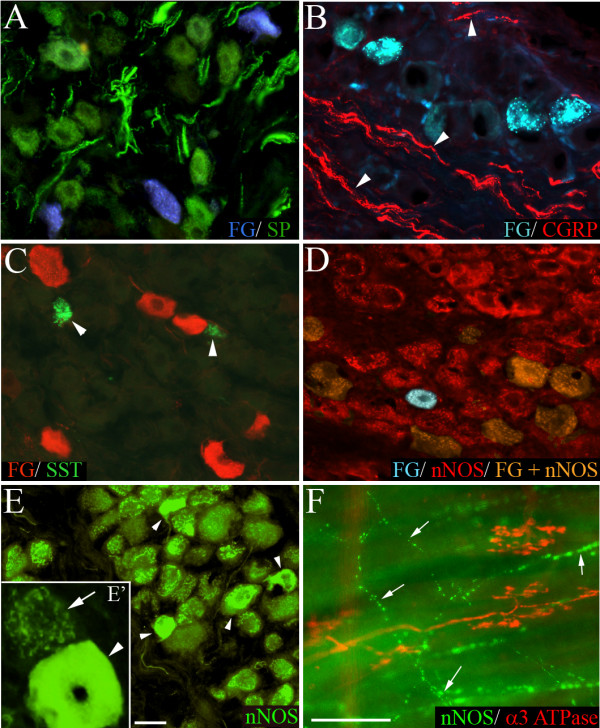
**Representative photomicrographs showing nodose neurons retrogradely labeled from the trachea with fluorogold (FG) overlaid with immunoreactivity for (A) substance P (SP), (B) calcitonin gene-related peptide (CGRP), (C) somatostatin (SST), and (D) neuronal nitric oxide synthase (nNOS).** The arrow heads in panels (B) and (C) point out CGRP-labeled nerve fibers and SST-labeled neurons, respectively. Panel (E) shows low and higher (E') magnification of nNOS immunoreactive cells in the nodose ganglia without FG overlaid. Note the clustered labeling associated with most neurons (arrow, E') and smaller population of intensely fluorescent cells (arrow heads, E and E'). In tracheal wholemounts (F), cough receptors identified with α3 Na+/K+ ATPase were not immunoreactive for nNOS, whereas fine varicose fibers (arrows) were nNOS positive (representative of 4 similar experiments). Traced neurons appear red in panel (C) as the tissue underwent secondary immunoprocessing for FG. Scale bar in panel E represents 50 μm in panels (A-E) and 20 μm in panel (E'). Scale bar in panel (F) represents 50 μm.

Immunoreactivity for the neuropeptides substance P, CGRP and somatostatin was almost exclusively confined to smaller neurons in the nodose ganglia (mean perimeters of untraced neurons were 99.1 ± 1.7, 90.0 ± 2.6 and 80.4 ± 2.2 for substance P, CGRP and somatostatin, respectively; P < 0.05 significantly smaller than the mean perimeter of fluorogold traced neurons) (Fig 6A). Substance P was present in both soma and nerve fibers throughout the nodose ganglia, whereas CGRP was restricted to nerve fibers (substantially fewer cells were immunoreactive for this peptide) (Fig 7). Somatostatin immunoreactivity was extremely sparse in both soma and fibers and, when seen, was often confined to very small neurons (Fig 6A and Fig 7). Of the fluorogold traced neurons, 8.7 ± 2.1 per cent (22 out of 260 traced neurons, n = 4 animals) expressed detectable levels of substance P, less than 1 per cent expressed CGRP (1 out of 210 traced neurons) and none expressed somatostatin (Fig 3 and Fig 7). The small population of substance P-positive, fluorogold-positive neurons identified in the nodose ganglia were significantly smaller in size compared to the larger population of neuropeptide-negative fluorogold traced neurons in this ganglia (P < 0.05, Table 2).

Immunoreactivity for nNOS was observed in many neurons in the nodose ganglia, albeit with two quite distinct patterns of expression. Most nodose neurons exhibited nNOS immunoreactivity that was characterized by numerous distinct dense fluorescent clusters throughout the cytoplasm (Fig 7E). By contrast, less than 10 per cent of the nNOS positive neurons showed more uniform intensely fluorescent cytoplasmic labeling (Fig 7E). The cells that exhibited clustered labeling and the intensely fluorescent cells (IFCs) largely shared overlapping somal size distributions (Fig 6B), although the nNOS IFCs were generally slightly smaller (112.6 ± 2.9 versus 98.3 ± 2.6 μm for the cells with clustered labeling and IFCs, respectively; Table 2). Most (> 90 per cent) of the fluorogold-traced neurons showed detectable immunoreactivity for nNOS (Fig 3 and 7D). However, only 2.8 ± 0.7 per cent of traced neurons were nNOS IFCs (Fig 3) and these cells were significantly (P < 0.05) smaller in size compared to the remainder of the fluorogold-traced neurons (Table 2). Immunoreactivity for nNOS was not observed in cough receptor nerve terminals in the tracheal submucosa (identified using α3 Na+/K+ ATPase wholemount immunohistochemistry; [6]) but rather was expressed in a subset of varicose nerve fibers (Fig 7F), resembling those fibers immunoreactive for substance P (Fig 1).

### Immunohistochemical expression of cytosolic proteins in nodose ganglia

**Figure 8:**
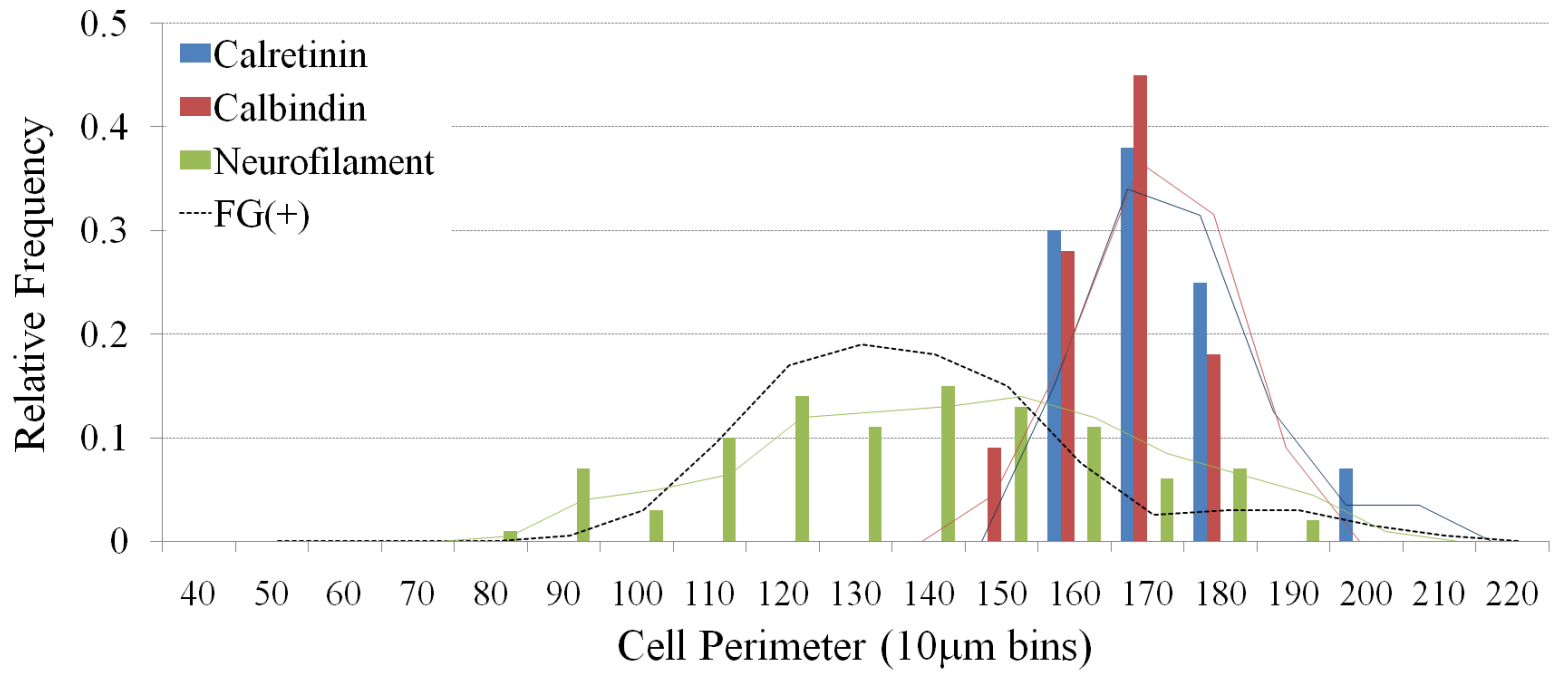
**Histogram showing the size distribution of all nodose neurons (irrespective of fluorogold tracing) that express calretinin, calbindin or neurofilament.** The superimposed solid lines show the moving averages associated with each histogram and the dashed line references the size distribution of fluorogold (FG) traced neurons shown in figure 2. Parvalbumin is not shown as there were no neurons expressed this marker.

**Figure 9:**
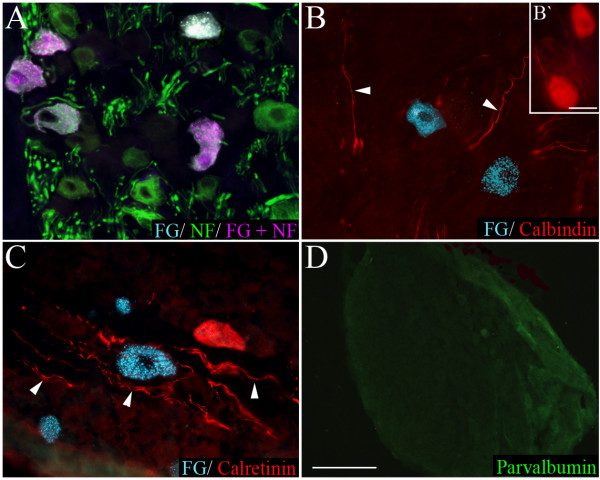
**Representative photomicrographs showing nodose neurons retrogradely labeled from the trachea with fluorogold (FG) overlaid with immunoreactivity for (A) neurofilament (NF), (B) calbindin, (C) calretinin, and (D) parvalbumin (low magnification showing no immunostaining).** The arrow heads in panels (B) and (C) point out calbindin and calretinin-labeled nerve fibers, respectively. The inset (B') shows two nodose neurons from an adjacent region of the ganglia that were immunoreactive for calbindin. The scale bar in (B') represents 50 μm. The scale bar shown in panel D represents 50 μm in panels (A-C) and 250 μm in panel (D).

As previously reported [2,11], neurofilament immunoreactivity in the nodose ganglia was observed in many medium and large sized neurons (Fig 8 and Fig 9A). Calretinin and calbindin immunoreactivity in the nodose was confined to nerve fibers and a relatively small number of large sized cells (150–200 μm) (Fig 8 and Fig 9B, C). Parvalbumin immunoreactivity (Fig 9D) was not present in any nodose structures (although was observed in neurons and nerve processes in the guinea pig brainstem, confirming that the antisera employed is appropriate for guinea pig tissues, data not shown).

Approximately 90 per cent of the neurons retrogradely labeled with fluorogold expressed neurofilament (Fig 3 and Fig 9A). By contrast there were no fluorogold-positive neurons that exhibited either calretinin or calbindin (or parvalbumin) immunoreactivity (Fig 3 and Fig 9B–D). The population (approximately 10 per cent) of fluorogold-positive neurofilament negative neurons were significantly (P < 0.05) smaller in size compared to the traced neurons that were neurofilament-positive (Table 2).

## Discussion

In the present study we investigated the expression of a variety of neurochemical markers in cough receptor neurons in the nodose ganglia. Retrograde neuronal tracing from the airways confirmed previous studies showing that the majority of nodose neurons projecting to the trachea have medium somal sizes and express neurofilament, a marker for myelinated neurons [2,11,12]. The minor population of small sized neurons that were retrogradely labeled did not express neurofilament, but rather stained positively for neuropeptides such as substance P or CGRP. All traced neurons in the nodose ganglia expressed the Na+/K+/2Cl- co-transporter, NKCC1. By contrast, although many medium sized traced neurons (cough receptor neurons) expressed α1 or α3 Na+/K+ ATPase, vGlut1 or vGlut2, none of these markers were universally expressed by all cough receptor cells. Most neurons in the nodose ganglia displayed detectable levels of nNOS immunoreactivity. However, intense immunolabeling for nNOS was not characteristic of cough receptor neurons and nNOS was not observed in cough receptor nerve terminals in the tracheal wall. Furthermore, cough receptors did not express somatostatin, calretinin, calbindin or parvalbumin. These data provide a detailed immunohistochemical characterization of guinea pig cough receptor neurons in the nodose ganglia. Furthermore our data suggest that, despite the evidence suggesting homogeneity in their peripheral physiology, it is likely that variations exist in the neurochemical profile of some cough receptors.

### Characterization of cough receptors in guinea pigs

Previous studies have characterized a novel airway afferent nerve subtype in guinea pigs that appears to be essential for defensive cough in this species [[1,7], reviewed in [4]]. These cough receptors represent a subset of mechanically sensitive afferent nerves innervating the extrapulmonary airways. This distribution (at least in guinea pigs) is in contrast to the terminal location of the classically defined rapidly and slowly adapting receptors (RARs and SARs) which are mainly confined to the intrapulmonary airways and lungs. Cough receptors also display very distinct activation profiles and electrophysiological properties compared to RARs and SARs [1]. Cough receptors are readily differentiated from bronchopulmonary C-fibers by their lack of sensitivity to capsaicin and bradykinin, faster conduction velocity and lack of expression of substance P and TRPV1 [1,2,13], and from the vagal afferents that innervate neuroepithelial bodies (NEBs) by their terminal locations (sub-epithelial, rather than associated with specialized epithelial cells, and exclusively extrapulmonary) [14]. Guinea pigs also have reportedly very few NEBs [15].

The available electrophysiological data suggests that almost all of the nodose neurons projecting to the guinea pig trachea display activation profiles that classify them as cough receptors [1-3,13,16-18]. Few capsaicin-sensitive airway afferents arising from the nodose ganglia innervate the guinea pig trachea (most originate from the jugular ganglia) and in guinea pigs the mechanically-sensitive nodose nerve endings in the trachea don't display the characteristics of RARs or SARs (although other species such as dogs and rabbits possess RARs and/or SARs in the trachea) [1,2,19,20]. There is also no evidence to suggest that individual cough receptors vary significantly in their basic physiology. The available anatomical data would also support the assertion that nodose neurons innervating the trachea are relatively homogenous. These observations make guinea pigs an ideal species for characterizing tracheal cough receptors. Thus, retrograde labeling of tracheal afferent nerves in the guinea pig nodose ganglia reveals a major population (95–99%) of medium to large sized neurons that do not express substance P or TRPV1 (markers used to define capsaicin-sensitive nociceptors) but do express neurofilament proteins (a marker for myelinated axons) [2,11,12]. These neurons are presumably the cough receptor neurons that have been identified functionally. The minor population of nodose neurons that project to the trachea (~5%) show the characteristics of small, unmyelinated nociceptors. Our data are consistent with these observations.

The results of the present study, however, provide some evidence that not all cough receptor neurons are identical. Not all neurons in the nodose ganglia that were retrogradely labeled from the trachea expressed α1 or α3 Na+/K+ ATPase, vGlut1 or vGlut2. Unlike the neurofilament negative traced neurons which displayed a significantly smaller somal size compared to the neurofilament positive traced neurons (suggesting that they are small diameter nociceptors), the average somal sizes of the retrogradely labeled neurons that were negative for α1 and α3 Na+/K+ ATPase, vGlut1 and vGlut2 immunoreactivity were not significantly different to the size of cough receptor neurons. This would suggest that some cough receptors likely differ in their expression of certain neuronal markers. A similar observation has been made with respect to the myelinated vagal afferent nerves that innervate pulmonary NEBs in rats (two myelinated vagal afferent types can be differentiated by the expression of α3 Na+/K+ ATPase, and P2X3 receptors) [21]. Whether clearly definable and distinct subsets of cough receptor neurons can be differentiated based on these, or other neuronal markers, awaits additional analyses.

### NKCC1 and Na+/K+ ATPase expression by cough receptors

The results from the present study confirm our previous data which showed that nodose neurons and cough receptor nerve terminals in the airways express NKCC1 [6], although NKCC1 is not a specific marker for cough receptor neurons as seemingly all neurons in the nodose ganglia showed NKCC1 labeling. NKCC1 functions to accumulate intracellular chloride ions above the electrochemical equilibrium in somatic (and likely vagal) sensory neurons [6,22,23], allowing a depolarizing chloride current to contribute to the regulation of afferent activity. Depolarizing chloride currents in sensory neurons are particularly critical to GABA-evoked primary afferent depolarization and inhibition of neurotransmitter release from the central projections of somatic and vagal sensory terminals in the spinal cord and brainstem (reviewed in [24]). More recently, NKCC1-mediated chloride uptake has also been suggested to be an important mechanism regulating the peripheral excitability of sensory neurons [6,23,25] and may represent a useful peripheral target for suppressing afferent nerve excitability [6,23,26].

Unlike NKCC1 expression, populations of sensory neurons may be differentiated based on the sodium pump isozyme which they express. For example, previous studies have revealed that in the dorsal root ganglia (DRG) α1 Na+/K+ ATPase is commonly expressed across many DRG neurons of varying somal sizes whilst α3 Na+/K+ ATPase is apparently restricted to medium and large sized neurons that presumably give rise to mechanically sensitive afferent fibers [27,28]. This is in agreement with our data in the nodose ganglia showing that α3 Na+/K+ ATPase, but not α1 Na+/K+ ATPase, immunolabeling is restricted to medium and large sized neurons. In the airways, α3 Na+/K+ ATPase is not expressed in substance P-containing C-fibers (Fig 1 of present study; [6]) but is present in the peripheral terminals of SARs in rabbits and rats [21,29], myelinated afferent fibers associated with NEBs in rats [21] and cough receptor terminals in guinea pigs (Figs 1 and 7 of present study; [6,30]). Moreover, few neurons in the jugular ganglia that project to the trachea (exclusively C- and Aδ-fiber nociceptors; [1,2]) express α3 Na+/K+ ATPase (S.B. Mazzone and A.E. McGovern, unpublished data). There have been no previous reports of α1 Na+/K+ ATPase expression in vagal afferent nerves, but our data suggest that more than half of the cough receptors in the nodose ganglia express detectable levels of α1 Na+/K+ ATPase immunoreactivity. It is not known what proportion of cough receptors express one versus both isozymes of Na+/K+ ATPase.

The apparent selective expression of α1 or α3 containing isozymes of Na+/K+ ATPase in different sensory neurons raises the question of what specific contribution the various sodium pump isozymes have on sensory neuron function. The more ubiquitous expression of α1 Na+/K+ ATPase across neurons of all sizes may suggest that pump isozymes containing this subunit play more of a housekeeping role in regulating sensory neuron Na+ and K+ gradients [31]. By contrast, some evidence suggests that α3 Na+/K+ ATPase may be specialized to mechanoreceptors. Indeed, the Na+/K+ ATPase inhibitor ouabain, at doses that are reportedly selective for inhibiting the α3 subunit, inhibits cough receptor activation and coughing evoked by citric acid, mechanical stimulation or electrical stimulation of the tracheal mucosa in anesthetized guinea pigs while having no effect on C-fiber dependent reflexes evoked from the trachea [30]. This effect may be due to unique kinetic properties of the α3 Na+/K+ ATPase isozyme that facilitate high frequency action potential conduction along the cough receptor axons [30]. Experiments investigating the high frequency firing properties of rat calyx of Held nerve terminals also support this assertion [32]. However, it is not known if the population of nodose mechanoreceptors that lack α3 Na+/K+ ATPase (present study) display different physiological attributes. It is also worth adding that Na+/K+ ATPase activity may be intrinsically linked to NKCC1 in cough receptors. NKCC1 would presumably elevate intracellular sodium ion concentrations (in addition to chloride) thereby facilitating Na+/K+ ATPase activity.

### Cough receptor neurotransmitters

Although a rigorous analysis of the neurotransmitters expressed by cough receptors was not conducted, several points are worthy of note. The expression of vGlut1 and/or vGlut2 by cough receptor neurons would suggest that cough receptors utilize glutamate as a key neurotransmitter. The vGluts are a family of transporters that are responsible for packaging glutamate into synaptic vesicles, and a substantial body of evidence shows that these proteins are reliable markers for glutamatergic neurons (e.g., [33]). Consistent with this, cough receptor evoked coughing in rabbits and guinea pigs is inhibited by selective ionotropic glutamate receptor antagonists injected in to the nucleus of the solitary tract [34,35].

In line with previous studies, our experiments have also failed to identify any neuropeptides in healthy cough receptor neurons. Studies to date have shown convincingly that cough receptors do not normally express substance P, CGRP, somatostatin or dynorphin [present study; [1,11,12,36]]. However, this is not to say that cough receptors don't contain a yet to be identified neuropeptide, as many neurons utilize neuropeptides as co-transmitters in the mammalian nervous system. The present data also suggest that cough receptor neurons may not use nitric oxide as a neurotransmitter. However, the results from these experiments are more difficult to interpret. In our hands, nNOS immunoreactivity in nodose ganglia neurons showed two distinct patterns: many cells possessed dense nNOS immunoreactive clusters, whereas substantially fewer cells displayed uniform intense somal labeling. Importantly, very few nodose neurons that projected to the airways showed intense uniform immunoreactivity for nNOS (those that did tended to be smaller cells, probably nodose nociceptors). Similar results have been previously reported in guinea pigs [37]. Moreover, nNOS was not present in cough receptor terminals, but was expressed by fine varicose fibers, in the guinea pig trachea. Nevertheless, given that nNOS expression was not assessed in the central terminals of cough receptors we cannot conclude definitively that NOS is not a neurotransmitter of cough receptors. The exact nature of the clustered nNOS immunoreactivity is also unclear at present. It may represent labeling of non-neuronal structures (e.g., glia associated with nodose neurons), labeling of nNOS in specific cellular compartments that have no relation to neurotransmission, or non-specific labeling produced by the antisera. It is, however, interesting that nitric oxide has been shown to mediate inter-somal transmission in the guinea pig nodose ganglia, suggesting the existence of a specific source of NOS for generating releasable nitric oxide [38].

### Absence of calcium binding proteins in cough receptors

A final interesting observation to arise from the present studies was the distinct lack of expression of calretinin, calbindin or parvalbumin in nodose cough receptor neurons. This is in contrast to studies of other vagal mechanosensitive and/or myelinated afferent nerve populations. In rats, the vagal afferents that innervate NEBs are defined in part by their expression of calbindin [39]. Furthermore, pulmonary smooth muscle associated receptors (likely SARs) and laryngeal mechanosensors express calretinin in rats [21,40,41], and parvalbumin and calbindin have been shown to be reliable markers for a variety of other vagal (or other visceral) mechanosensory neurons [42-44]. In our studies, calretinin and calbindin only labeled a subset of very large neurons in the nodose, whereas parvalbumin failed to label any structures in the nodose ganglia (despite labeling neurons and fibers in the guinea pig brainstem, not shown). Whether the large neurons immunoreactive for calbindin and calretinin consist in part of mechanoreceptive neurons that project to the intrapulmonary airways (i.e., RARs and/or SARs) is unknown, but may reveal an alternative method for differentiating cough receptors from other airway mechanosensors.

In summary, the present data provide further characterization of the nodose neurons that innervate the guinea pig trachea and suggest that immunohistochemically distinct subtypes of presumed cough receptors likely exist.

## List of abbreviations

CGRP: Calcitonin Gene-Related Peptide; DRG: Dorsal Root Ganglia; IFCs: Intensely Fluorescent Cells; NEB: Neuroepithelial Body; NKCC1: Na+/K+/2Cl- Co-transporter 1; nNOS: neuronal Nitric Oxide Synthase; RAR: Rapidly Adapting Receptor; SAR: Slowly Adapting Receptor; TRPV1: Transient Receptor Potential Vanilloid 1; vGlut: vesicular Glutamate transporter

## Competing interests

The authors declare that they have no competing interests.

## Authors' contributions

AM carried out the immunohistochemical experiments, performed some data analysis and drafted some sections of the manuscript. SM conceived and designed the study, analysed some data and wrote and compiled the manuscript. All authors read and approved the final manuscript.
